# Encapsulated peracetic acid as a valid broad-spectrum antimicrobial alternative, leading to beneficial microbiota compositional changes and enhanced performance in broiler chickens

**DOI:** 10.1186/s40104-023-00881-w

**Published:** 2023-06-09

**Authors:** Salvatore Galgano, Leah Conway, Nikki Dalby, Adrian Fellows, Jos G. M. Houdijk

**Affiliations:** 1 Monogastric Science Research Centre, Scotland’s Rural College, West Mains Road, Edinburgh, EH9 3JG Scotland UK; 2 Aga2tech Ltd, Halifax, UK; 3 Centre for Innovation Excellence in Livestock, York, UK

**Keywords:** Antimicrobial alternative, Antimicrobial resistance, Broiler chicken, Microbiota, Peracetic acid, Performance, 16S rRNA gene

## Abstract

**Background:**

Antimicrobial alternatives are urgently needed, including for poultry production systems. In this study, we tested the potential broad-range antimicrobial alternative peracetic acid, delivered in feed via the hydrolysis of encapsulated precursors through a 28-day study using 375 Ross 308 broiler chickens. We tested two peracetic acid concentrations, 30 and 80 mg/kg on birds housed on re-used litter, and we evaluated the impact of both levels on gut microbial communities, bacterial concentration, antimicrobial resistance genes relative abundance and growth performance when compared to control birds housed on either clean or re-used litter.

**Results:**

Body weight gain and feed conversion ratio improved in peracetic acid fed birds. At d 28, birds given 30 mg/kg of peracetic acid had a decreased Firmicutes and an increased Proteobacteria abundance in the jejunum, accompanied by an increase in *Bacillus*, *Flavonifractor* and *Rombustia* in the caeca, and a decreased abundance of tetracycline resistance genes. Chicken given 80 mg/kg of peracetic acid had greater caecal abundance of macrolides lincosamides and streptogramins resistance genes. Growth performance on clean litter was reduced compared to re-used litter, which concurred with increased caecal abundance of *Blautia*, decreased caecal abundance of *Escherichia/Shigella, Anaerostipes* and *Jeotgalicoccus*, and greater gene abundance of vancomycin, tetracycline, and macrolides resistance genes.

**Conclusions:**

Peracetic acid could be used as a safe broad-spectrum antimicrobial alternative in broilers. Encapsulated precursors were able to reduce the bacterial concentration in the jejunum whilst promoting the proliferation of probiotic genera in the caeca, especially at the low peracetic acid concentrations tested, and improve growth performance. Moreover, our findings offer further insights on potential benefits of rearing birds on re-used litter, suggesting that the latter could be associated with better performance and reduced antimicrobial resistance risk compared to clean litter rearing.

**Supplementary Information:**

The online version contains supplementary material available at 10.1186/s40104-023-00881-w.

## Background

Antimicrobial resistance (AMR) can occur intrinsically amongst microorganisms or develop through adaptive mechanisms that could selectively lead to the acquisition of new resistance genes either via mutations or by obtaining external DNA [1]. Multi-drug resistant organisms (MDRO) are commonly associated with livestock, including in poultry production systems, and can represent a reservoir-risk for humans due to both their residual presence in meat and their environmental spread [2]. Indeed, AMR abundance amongst pathogens, translates to a decreased treatment availability for the patients, leading to growing morbidity and mortality [3], aided by the escalating development of AMR in pathogens, also driven by misuse of antimicrobials in livestock production systems [4]. It is now well established how the selective pressure operated by antibiotic administration, especially low-dose ones used in the past as growth promoters, is directly connected to a rise in AMR in poultry production [5]. Even though low-dose antibiotics have been administered for several years to broiler birds as growth promoters, their precise mechanism of action towards increased performance has never been conclusively elucidated [6], before being banned in 2006 in Europe and other parts of the world [7]. Several antibiotic alternatives have been tested and proposed for some time, such as probiotics, prebiotics, organic acids, and plant extracts [8], together with more recently cell-free supernatant [9], bacteriophages [10] and non-organic molecules [11]. The modulation of the chicken gut microbiota ought to be the focal point of any of these approaches [12], as the final effect of any antibiotic alternative is due to adaptation mechanisms and resilience of the gut symbionts upon the initial perturbation caused by the intervention.

Peracetic acid (PAA) is a powerful oxidative agent with broad spectrum antimicrobial activity [13], whose degradation does not lead to harmful by-products [14]. In this study, we delivered PAA in vivo in broilers via hydrolysis of precursors sodium percarbonate (SP) and tetraacetylethylenediamine (TAED), which leads to the formation of peracetic acid and hydrogen peroxide as biocompatible compounds. To ensure delivery to the more distal part of the gastrointestinal tract, both SP and TAED were encapsulated in Eudragit™ ammonio methacrylate copolymer type A (RLPO) polymer, whose efficacy as drug delivery system has already been proven in humans [15, 16].

Currently, poultry bedding (litter) management differs according to internal regulations to each country or continent. In Europe for example, litter is changed, and houses are disinfected after each production cycle, whereas in the USA litter is used for several consecutive growth cycles [17]. Accumulating evidence points towards the importance of the interactions between litter and chicken microbiota [18]. We tested five experimental conditions, which included three controls, i.e., clean litter and no PAA (treatment code: CLC), re-used litter and no PAA (treatment code: RLC), and re-used litter and empty RLPO particles (0 mg/kg, treatment code: 0 ppm, i.e., no PAA generated) and two PAA levels of inclusion, 30 mg/kg (treatment code: 30 ppm) and 80 mg/kg (treatment code: 80 ppm), each in the presence of reused litter.

To the best of our knowledge the effects of PAA administration to broiler chickens through hydrolysis of feed-added encapsulated precursors on microbiota, performance and main AMR gene abundance have not been assessed. The results presented here suggest that PAA is safe to use in broilers and dose specific, with 30 mg/kg showing better results both in terms of effect on performance and decreased risk of AMR passive selection. Moreover, we found that the use of re-used litter may improve performance and reduce AMR selection risk.

## Materials and methods

### Animal study and treatment preparation

A 28-day animal study was carried out at SRUC’s Allermuir Avian Innovation and Skills Centre, where a total of 375 day-old Ross 308 male broilers were randomly allocated to 75 pens, with 15 pen-replicates per treatment and 5 birds per pen. A total of 5 experimental conditions were tested, with three controls and two PAA treatments (Table 1). The study followed a randomised block design with 4 rooms and a total of 15 blocks (5 pens per block). A total of 20 pens (4 blocks) were allocated in the first 3 rooms and 15 pens (3 blocks) were allocated in the last room. Bespoke commercial diets (see Additional file 1) for the starter phase (d 0–14) and grower (d 14–28) phases were both offered as mash and provided ad-libitum to the birds, formulated to meet standard nutrient requirements (crude protein: ~ 23%, 2,800 kcal metabolizable energy/kg).

**Table 1:** Treatment description and code

	Clean litter control	Re-used litter control	Empty RLPO capsules control	RLPO-encapsulated PAA (30 mg/kg)	RLPO-encapsulated PAA (80 mg/kg)
Code	CLC	RLC	0 ppm	30 ppm	80 ppm
Reused litter	No	Yes	Yes	Yes	Yes
RLPO mixed with feed			0.66%	0.412%	
RLPO-TAED mixed with feed				0.124%	0.33%
RLPO-SP mixed with feed				0.124%	0.33%

*PAA* Peracetic acid, *ppm* Parts per million, *RLPO* Eudragit™ ammonio methacrylate copolymer type A, *SP* Sodium percarbonate, *TAED* Tetraacetylethylenediamine

PAA was delivered via hydrolysis of encapsulated precursors SP and TAED, whose formulation was manufactured by Aga2tech ltd, Halifax, UK. The mixing of RLPO-TAED and RLPO-SP at specific ratios with feed was done to achieve either 30 mg/kg or 80 mg/kg of PAA following precursor hydrolysis in vivo (Table 1). The 0 ppm control (RLPO only) was prepared using the same absolute particle concentration as found in the 80 ppm treatment, to reduce the bias due to the administration of different ratios of starting material. RLC, 0 ppm, 30 ppm and 80 ppm birds were housed on re-used litter from the control group of a preceding nutrition study with slow growing birds, according to a 90:10 ratio with fresh bedding, whereas 100% of the latter was used for the CLC group. Study design and protocol were approved by SRUC Animal Welfare and Review Body (POU AE 16–2021).

### Sample collection, pH measurement and DNA isolation

At d 28, 2 birds per pen were dissected after being humanely culled via cervical dislocation and the content of crop, gizzard, jejunum, ileum, caeca was collected and immediately stored at −78 °C (dry ice) for downstream DNA isolation as specified below. The colon content was also collected and immediately used for pH measurement (1111105 2-star benchtop pH meter, Thermo Scientific, Waltham, MA, USA), together with an aliquot of ileum and caecal content. Samples for DNA isolation (0.25 g) were collected in PowerBead Pro Tube of the QIAsymphony PowerFecal Pro DNA Kit (Cat. No. 938036, QIAGEN, Hilden, Germany) and transferred to a −80 °C ultra-low temperature freezer as soon as possible until DNA isolation was carried out at SRUC Biomarkers Lab (Edinburgh, UK) following manufacturer instruction on a QIAsymphony SP (Cat. No. 9001297, QIAGEN, Hilden, Germany). Thus, 4 µL of RNase A (Cat. No. 19101, QIAGEN, Hilden, Germany) were added to each sample after homogenization of the PowerBead Pro Tubes in a FastPrep-24TM 5G homogenizer (MP Biomedicals, Santa Ana, CA, USA) for 55 s at 5.5 m/s.

### Bacterial absolute quantification

Absolute qPCR quantification was carried out based on a nine-point standard curve built via ten-fold serial dilutions of linear plasmid [19] containing the 16S V3 region [20] amplicon target as insert (341F: 5’-CCTACGGGAGGCAGCAG-3’, 518R: 5’-ATTACCGCGGCTGCTGG-3’), as same target used through the qPCR. Gel-purified insert-PCR product (Wizard® SV Gel and PCR Clean-Up System, Promega, Madison, WI, USA) was cloned into a pCR2.1 plasmid (TA Cloning™ Kit, Thermo Fisher Scientific, Waltham, MA, USA) and transformed into competent One shot® INVαF’ *E. coli* cells (Thermo Fisher Scientific, Waltham, MA, USA) by heat shock. Plasmid was isolated from positive colonies in Luria Bertani broth (QIAprep Miniprep kit, QIAGEN, Hilden, Germany) and insert presence was verified by both *EcoRI* (New England BioLabs, Ipswich, MA, USA) digestion and by Sanger sequencing (DNA Sequencing and Services, Medical Sciences Institute, School of Life Sciences, University of Dundee), before linearization with 5 units of *HindIII* (New England BioLabs, Ipswich, MA, USA). Absolute qPCR quantification was carried out in a Mx3000 thermocycler (Agilent Technologies, Santa Clara, CA, USA) at 95 °C for 3 min followed by 40 cycles at 95 °C for 10 s and 60 °C for 20 s, at the end of which SYBR green fluorescence was detected. Reactions (20 µL) included 1 ng of DNA template, 1× Brilliant III Ultra-Fast SYBR Green qPCR Master Mix (Agilent technologies, Santa Clara, CA, USA) and 200 nmol/L of each primer. All the reactions were run in triplicate including a non-template control, whilst quality was assessed both by melting curve and standard curve analysis, with average values for R^2^, slope and efficiency as 0.99, −3.3 and 100%, respectively considered satisfying.

Concentration of bacteria per gram of gut content was calculated from copy number (CN) per reaction output of the above. Firstly, number of bacteria per reaction was calculated via dividing qPCR-CN by 5.2, as the average 16S gene CN per bacterial cells at the time of writing [21]. Thus, the luminal bacterial concentration was calculated as previously described [22].

### 16S gene sequencing and bioinformatic analysis

One bird from a total of 7 pens per treatment was selected for microbiota analysis of crop, jejunum, and caeca content. Pens were selected based through the following procedure. Pens were ranked according to their body weight (BW) at d 28 for each treatment with 1 being the highest BW and 15 the lowest, respectively. Then, after excluding the pens ranked 1^st^ and 15^th^, 7 groups of pens were formed (Table 2), and the gut-content DNA of the bird with the BW closer to the average of the group BW was used.

**Table 2:** Sample selection rationale for sequencing analysis

Pens ranked according to BW at d 28	Group of ranked pens
1	
2	1
3	2
4	
5	3
6	
7	4
8	
9	
10	5
11	
12	6
13	
14	7
15	

Pens were ranked according to their descending body weight at d 28. Thus, 7 groups were formed, and the 7 samples were selected from each group excluding samples from pen ranked in 1^st^ and 15^th^ position

16S rRNA gene sequencing was carried out by Omega Bioservices (Norcross, GA, USA), together with library preparation targeting the V4 region of the bacterial 16S rRNA gene (F515_b_ [23]: 5'-TCGTCGGCAGCGTCAGATGTGTATAAGAGACAGGTGYCAGCMGCCGCGGTAA-3'; R806_b_ [24]: 5'-GTCTCGTGGGCTCGGAGATGTGTATAAGAGACAGGGACTACNVGGGTWTCTAA-3'). In short, amplicon PCR (total volume of 25 µL) was performed through 95 °C for 3 min followed by 25 cycles at 95 °C for 30 s, 55 °C for 30 s and 72 °C for 30 s, with further final 5 min at 72 °C, and components (final concentration) were, 12.5 ng of template DNA, 1 × KAPA HiFi HotStart ReadyMix (Kapa Biosystems, USA) and 0.2 µmol/L of each primer. PCR product was purified (Mag-Bind RxnPure Plus magnetic beads, Omega Bio-tek, USA), before a second index PCR amplification, incorporating barcodes and adapters was performed, with same component concentrations as above at 95 °C for 3 min, 8 cycles of 95 °C, 55 °C and 72 °C, each for 30 s, and 5-min step at 72 °C. Quality control on the libraries (~ 600 bases) was performed with 2200 TapeStation (Agilent technologies, Santa Clara, CA, USA) and quantified using QuantiFluor dsDNA System (Promega, Santa Clara, CA, USA), which were thus normalized, pooled and sequenced (2 × 300 bp paired end read setting) on the MiSeq (Illumina, Santa Clara, CA, USA).

FASTQ paired end demultiplexed reads were imported in QIIME2 v2022.2 [25], joined via VSEARCH [26] with quality score of ~ 40 and after quality-filtered with minimum Phred score of 20 [27, 28]. Reads were thus denoised via Deblur [29] with trimming length set at 260 bp and diversity analysis was carried out on even sequence depth of 4,861, generating α-diversity indexes through richness and Shannon’s diversity [30, 31] and β-diversity matrixes through the Bray–Curtis dissimilarities and the Jaccard similarity index [32, 33]. Taxonomy was assigned with the q2-feature-classifier plugin with a Naïve Bayes classifier trained on the F515_b_/R806_b_ primers and the Silva data base (138, 99% of similarities) [34–36].

### AMR gene relative quantification

The caecal content of the one bird per pen, identified for total bacterial concentration and microbiome composition, was analyzed for AMR gene abundance. The relative abundance of a total of 20 AMR genes (i.e., *vanB, vanC, tetA, tetB, aadA1, aphA6, aacC4, mecA, SHV, CTX-M-1, CTX-M-9, OXA-48, ermA, ermC, msrA, QnrD, QnrB-8, aprm, ccrA* and *ereB*) was tested with in multiplex with the custom microbial DNA qPCR arrays (Cat. No. 330161, QIAGEN, Hilden Germany). The bespoke arrays included PanA/C, PanB1, PanB3, as microbial normalizers based on 16S sequences, allowing 2^−ΔΔCt^ analysis and a positive PCR control (PPC). To allow comparison between different plates through the same analysis, baseline (4^th^ to 14^th^ cycle) and threshold (0.1) settings were the same across all PCR arrays/runs, as per manufacturer instruction, and the cycle threshold (Ct) values for the PPC was 22 ± 2 across all arrays [37]. Reactions were carried out in 25 µL total, with, 12.5 µL of Master Mix and 0.735 ng of DNA template per reaction, thus thermal conditions were 95 °C for 10 min and 40 cycles of 95 °C for 15 s and 60 °C for 2 min, at the end of which FAM fluorescence was detected in a Stratagene Mx3000P (Agilent Technologies, Santa Clara, CA, USA) cycler.

Ct values output of the above were processed in the following way. Firstly, outliers (i.e., < µ − σ and > µ + σ) were excluded from the analysis, thus the average Ct of the normalizers (HK) was subtracted from the Ct of each AMR gene (ΔCt = Ct_AMR_ − Ct_HK_), therefore the ΔΔCt was calculated for each gene in each experimental condition compared to RLC and expressed as 2^−ΔΔCt^ used via statistical analysis as per below.

### Animal performance

Health evaluation of all the birds was carried out at d 0 before wing-tagging and individual BW and feed issued measurement. Thereafter, bird-level BW and both pen-level feed issued, and refusals were measured at d 7, 14, 21 and 28, allowing the calculation of both body weight gain (BWG) and mortality-corrected feed conversion ratio (MFCR) calculated as total feed intake per pen/[(pen BW + dead birds pen BW) – total pen BW at start of period]. Bird mortality was recorded daily throughout the study.

### Statistical analysis

Number of animals and pens used in this study (i.e., sample size) were determined prior to the animal trial via simulation-based power estimation through 102,000 simulations using the package SIMR [38] in R v4.1.2 [39] based on dataset stochastically generated starting from Ross 308 performance objectives [40]. Linear mixed model (LMM) was fitted in R using the package *lme4* [41], whilst LMM *P*-values were calculated in type III ANOVA via Satterthwaite's degrees of freedom method using the R package *lmerTest* [42]. LMM was fitted with longitudinal data, where possible, with treatment, and time (i.e., categorical fixed effects), and the hierarchy of room/block/pen/birds where possible, as random effects. The variables analyzed via LMM were log_10_ BWG, MFCR, AMR gene relative abundance, log_10_ bacterial concentration and both α-diversity indexes or richness and Shannon diversity, whereas statistical analysis on β-diversity indexes was calculated via permutational multivariate analysis of variance (PERMANOVA) [30]. Four linear (LMM) and two quadratic (ANOVA) contrast statements were also tested to assess the following specific comparisons: I) CLC vs. rest of the experimental conditions, to test for the effect of reused litter exposure per se, II) RLC vs. the combined 0 ppm, 30 ppm and 80 ppm treatments, to test for the effect of presence of RLPO particles per se, III) 0 ppm vs. the combined 30 ppm and 80 ppm PAA treatments, to test for the effect of PAA per se, IV) 30 ppm vs. 80 ppm, V) quadratic model with optimum at 30 ppm and VI) quadratic model with optimum at 80 ppm.

Differential abundance analysis was carried out on taxonomical output of the 16S analysis to assess specific compositional differences between experimental conditions, both at phylum and genus level. Microbiome Multivariable Association with Linear Models 2, MaAsLin2 [43] was used in R, where QIIME2-derived reads were normalized via total sum scaling (TSS) to eliminate sequencing depth-related bias and then log_10_ transformed, and the results were accompanied by both a *P* and a *Q* value to account for the positive false discovery rate. The package qiime2R [44] was used to graphically represent QIIME2 outputs of α- and β-diversity, including principal coordinate analysis (PCoA) for β-diversity.

## Results

### Impact of PAA on bacterial concentration

**Fig. 1:**
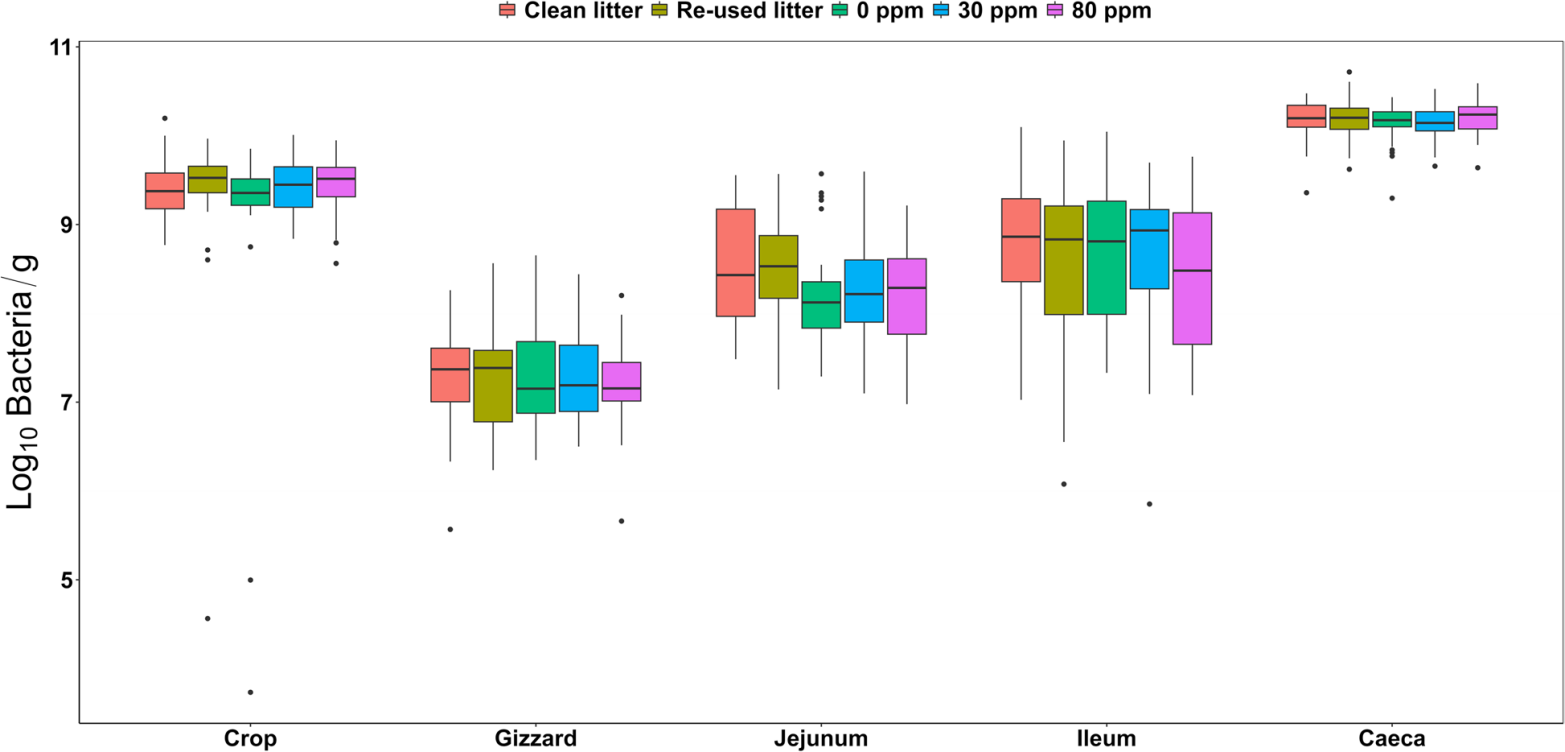
qPCR log10 abundance of the total number of bacteria, derived by the absolute quantification of the V3 copy number through the five experimental conditions

All the concentrations described below are all expressed in log_10_ bacteria/gram of gut content (Fig. 1). No differences were found in both caecal and ileal luminal bacterial concentration between the five experimental conditions, where the average log_10_ concentration was 8.6 ± 0.8 and 10.2 ± 0.2, respectively. However, contrast analysis revealed that both CLC (8.5 ± 0.7) and RLC (8.5 ± 0.6) jejunal bacterial concentration were greater compared to the PAA treatments (*P* < 0.05). Specifically, CLC had greater bacterial concentration than 0 ppm (8.2 ± 0.6, *P* = 0.06) and 80 ppm (8.2 ± 0.6, *P* < 0.05), whilst the latter was also smaller than RLC (*P* = 0.05). No significant differences were found between the bacterial concentration of the gizzard content between the different experimental conditions, where log_10_ concentration of CLC and RLC was 7.3 ± 0.6 and 7.4 ± 0.6, respectively, followed by 0 ppm (7.3 ± 0.6), 30 ppm (7.3 ± 0.5) and 80 ppm (7.2 ± 0.5). In the crop, and supported by the contrast statements, bacterial content in 0 ppm (9.0 ± 1.3) tended to be lower than 30 ppm (9.4 ± 0.3, *P* = 0.06), 80 ppm (9.4 ± 0.4, *P* = 0.07) and CLC (9.4 ± 0.3, *P* = 0.09), whereas RLC (9.3 ± 1) was intermediate and did not differ from all other experimental conditions.

### Effect of PAA on microbial composition through the different gut locations

A total of 20,869,644 FASTQ paired end demultiplexed reads, with ~ 200,000 reads/sample and 3,995 reads in the negative control, were joined and quality-filtered retaining 4,555,773 reads in total (~ 40,000 reads/sample and 144 reads/control). Trimming at 260 bp whilst denoising via Deblur allowed to use a sequencing depth of 4,861 features/sample, retaining 510,405 (25.92%) features in 105 (99.06%) samples (i.e., all but library-prep negative control). Only 8 reads in total were found in the library-prep negative control, with 50% belonging to Firmicutes and 50% to Cyanobacteria, of which 2 reads were aligned as *Lactobacillus*, 2 as Firmicutes CAG-56 and 4 were classified as Chloroplast*.*

**Fig. 2:**
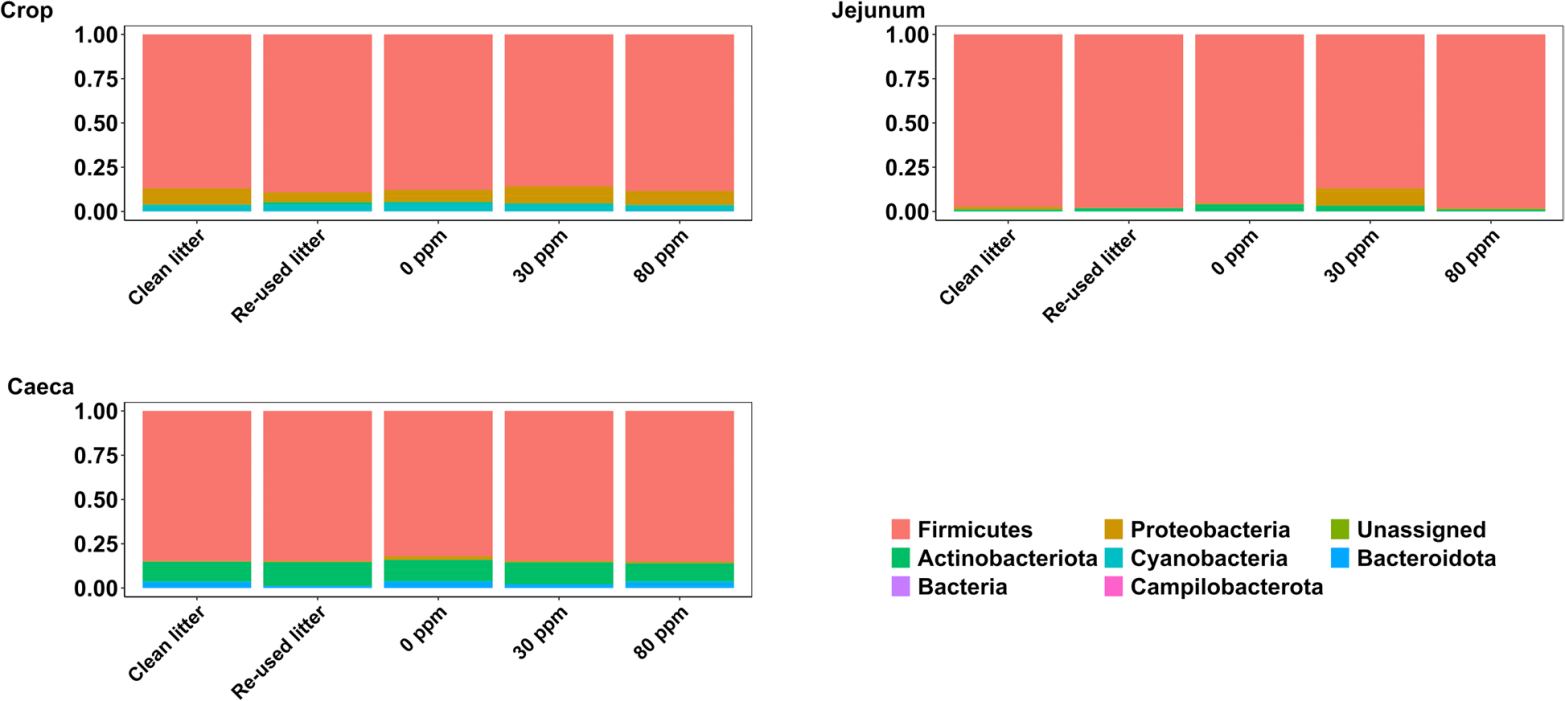
Phylum relative abundance in Crop, Jejunum and Caeca

**Fig. 3:**
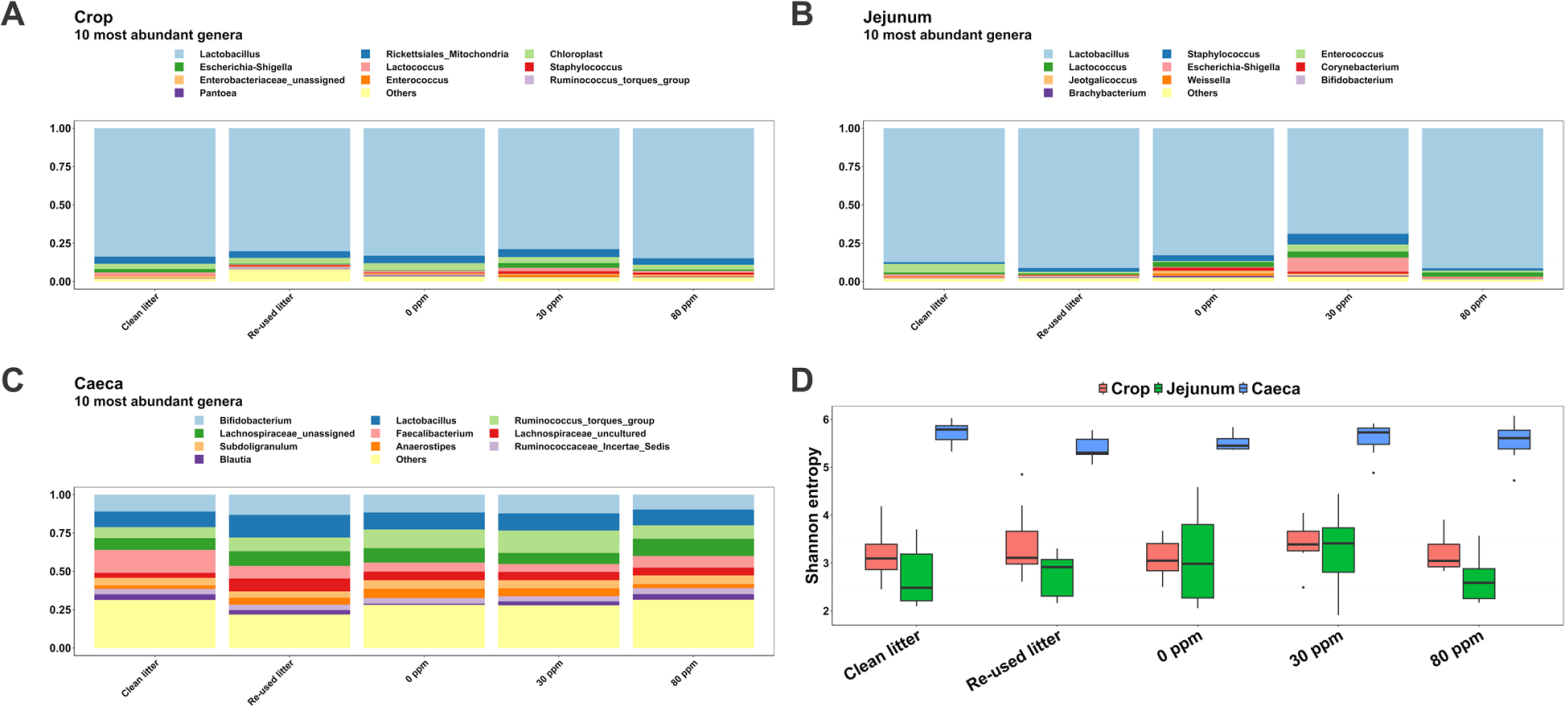
Relative abundance of the 10 most abundant genera in the lumen of crop (**A**), jejunum (**B**) and caeca (**C**). **D** Shannon index throughout the five experimental conditions (*x*-axis) and in the three different gut locations (different boxplot color)

A complete feature table, both at phylum and genus level, is provided within an additional file (see Additional file 2), whereas the most important differences between phyla, the five most abundant genera through the three gut locations and the five conditions are presented here. At phylum level (Fig. 2), Firmicutes was the most abundant in the crop (86.9% ± 6.2%), jejunum (95.2% ± 4.7%) and caeca (84.2% ± 4.9%), whilst taxonomical composition at genus level through the experimental conditions and three gut locations is depicted in Fig. 3A, B and C.

#### Crop

Proteobacteria was more predominant in CLC (10.1% ± 6.3%, *P* = 0.05, *Q* = 0.74) and in 30 ppm (10.7% ± 6%, *P* = 0.07, *Q* = 0.74) compared to RLC, 0 ppm and 80 ppm, whose relative abundance was 5.5% ± 1.9%, 6.9% ± 3.3% and 8.1% ± 5.6%, respectively. No evident changes through treatments were observed in Cyanobacteria, Actinobacteriota and Bacteroidota, whose average relative abundance was 3.8% ± 2.3%, 0.6% ± 0.8% and 0.2% ± 0.2%, respectively, whereas unassigned phyla in the crop were 0.1% ± 0.05% of the total across the five conditions. *Lactobacillus* was the most abundant genus in the crop in all experimental conditions (80.8% ± 11.7%), followed by *Escherichia/Shigella*, *Lactococcus* and *Staphylococcus* in CLC (2.7% ± 3.3%, 2.2% ± 3% and 0.4% ± 0.7%) and 30 ppm (4% ± 5.8%, 2.8% ± 3.1% and 2.3% ± 3.6%). *Staphylococcus* was the second most abundant genus in RLC (1% ± 1.3%), followed by uncultured *Lachnospiraceae* (0.9% ± 1.5%) and *Escherichia/Shigella* (0.6% ± 0.8%). Finally, *Lactococcus* was the second most abundant in 0 ppm (1.5% ± 2.4%) and 80 ppm (1.5% ± 2.8%) followed by *Staphylococcus* (0.7% ± 1% and 1.2% ± 2.2%) and *Escherichia/Shigella* (0.6% ± 0.9% and 0.9% ± 0.9%). Unassigned *Enterobacteriaceae* was less abundant in CLC compared to RLC (*P* < 0.05) and *Jeotgalicoccus* was decreased in CLC compared to 30 ppm and 80 ppm (*P* < 0.05, *Q* = 0.24).

#### Jejunum

Firmicutes in the 30 ppm group (89.9% ± 9.1%) was reduced compared to RLC (97.9% ± 2%, *P* < 0.05, *Q* = 0.05) and to CLC (96.7% ± 3.5%, *P* = 0.02, *Q* = 0.2), whilst Firmicutes relative abundance in 0 ppm and 80 ppm was 93.9% ± 7% and 97.6% ± 2%. Proteobacteria was the second most abundant phylum in both CLC (2% ± 2.9%) and 30 ppm (5.8% ± 8.6%), with the latter levels higher than RLC (0.2% ± 0.3%; *P* < 0.05, *Q* ≤ 0.05) and 0 ppm (0.6% ± 1%, *P* < 0.05, *Q* = 0.09). Proteobacteria abundance was greater for CLC than for RLC (*P* < 0.05, *Q* = 0.06), whilst Proteobacteria in 80 ppm was 0.9% ± 1.1%. Actinobacteriota was the second most abundant phylum in RLC (1.7% ± 1.7%), 0 ppm (5.3% ± 6.3%) and 80 ppm (1.3% ± 1%), whereas it was the third in abundance in 30 ppm (4.2% ± 5.1%) and CLC (1.2% ± 1.3%), followed by Cyanobacteria in RLC (< 0) and the rest of the conditions (~ 0.1% ±  ~ 0.1%).

*Lactobacillus* was the most abundant genus in RLC (90.9% ± 9.4%), CLC (83% ± 21.8%), 80 ppm (89.9% ± 10.6%), 0 ppm (78.5% ± 21.1%) and 30 ppm (68.2% ± 22.2%). In CLC, *Enterococcus* was the second most abundant genus (8.4% ± 15.2%), followed by *Escherichia/Shigella* (1.8% ± 2.9%), *Lactococcus* (1.6% ± 2%) and *Staphylococcus* (1.4% ± 1.8%). *Staphylococcus* was second most abundant in both RLC (2.5% ± 2.8%) and 0 ppm (5.3% ± 6.2%), followed by *Enterococcus* (1.5% ± 3.2%), *Lactococcus* (1% ± 0.8%) and *Brachybacterium* (0.3% ± 0.5%) in RLC, and *Lactococcus* (3% ± 3.2%), *Weissella* (2.3% ± 5.6%) and *Brachybacterium* (1.1% ± 1.4%) in 0 ppm. *Staphylococcus* was the second most abundant genus in 30 ppm (9.2% ± 9.2%), followed by *Enterococcus* (6.1% ± 5.8%), *Escherichia/Shigella* (5.5% ± 8.2%) and *Lactococcus* (2.9% ± 3%). *Lactococcus* was the second most abundant in 80 ppm (3.2% ± 5%), followed by *Staphylococcus* (1.8% ± 1.1%), *Enterococcus* (1.4% ± 3%) and *Escherichia/Shigella* (0.7% ± 0.9%). The latter was less abundant in RLC compared to 30 ppm (*P* < 0.05, *Q* = 0.07).

#### Caeca

The second most abundant phylum was Actinobacteriota (11.7% ± 4.8%), followed by Bacteroidota (2.8% ± 1.9%), Proteobacteria and Cyanobacteria in RLC (0.7% ± 0.6% and 0.01% ± 0.02%), 0 ppm (2.1% ± 1.3% and 0.6% ± 1.6%) and 30 ppm (0.9% ± 1.1% and 0.03% ± 0.08%). Cyanobacteria was more abundant than Proteobacteria in CLC (0.6% ± 1.4% and 0.3% ± 0.3%) and 80 ppm (1.1% ± 1.9% and 0.9% ± 0.3%). Caecal Proteobacteria in 0 ppm were more abundant than CLC (*P* < 0.01, *Q* = 0.02) and tended to be more abundant than both RLC (*P* = 0.01, *Q* = 0.09) and 30 ppm (*P* = 0.01, *Q* = 0.09).

The most abundant genus (Fig. 3C) was *Faecalibacterium* in CLC (14.4% ± 7.6%), *Ruminococcus* (*Torques* group) in 0 ppm (12.7% ± 6.1%) and 30 ppm (13.5% ± 9.5%), whilst *Lactobacillus* and unassigned *Lachnospiraceae* were most abundant in RLC (14.7% ± 4.6%) and in 80 ppm (11% ± 4.4%), respectively. *Bifidobacterium* was the second most abundant in all the experimental conditions (CLC, 10.8% ± 6%; RLC, 13% ± 3.9%; 0 ppm, 11.4% ± 5.4%; 30 ppm, 12.5% ± 2.7% and 80 ppm, 10.2% ± 6.1%), followed by *Lactobacillus* in CLC (10% ± 3%), 0 ppm (10.7% ± 4.3%), 30 ppm (10.8% ± 3.9%) and 80 ppm (9.4% ± 6.8%), and unassigned *Lachnospiraceae* in RLC (9.4% ± 3%). The latter ranked fourth in CLC (8.3% ± 5.9%), 0 ppm (9.5% ± 3.6%) and 30 ppm (7.2% ± 4.9%), together with *Ruminococcus* (*Torques* group) in RLC (9% ± 4%) and 80 ppm (9% ± 8.1%), followed by *Faecalibacterium* in 80 ppm (7.9% ± 6.4%), *Ruminococcus* (*Torques* group) in CLC (7.3% ± 5.8%), uncultured *Lachnospiraceae* in RLC (7.4% ± 5.5%) and in 30 ppm (9 5.8% ± 4.4%) and *Anaerostipes* in 0 ppm (6.3% ± 2.5%).

**Fig. 4:**
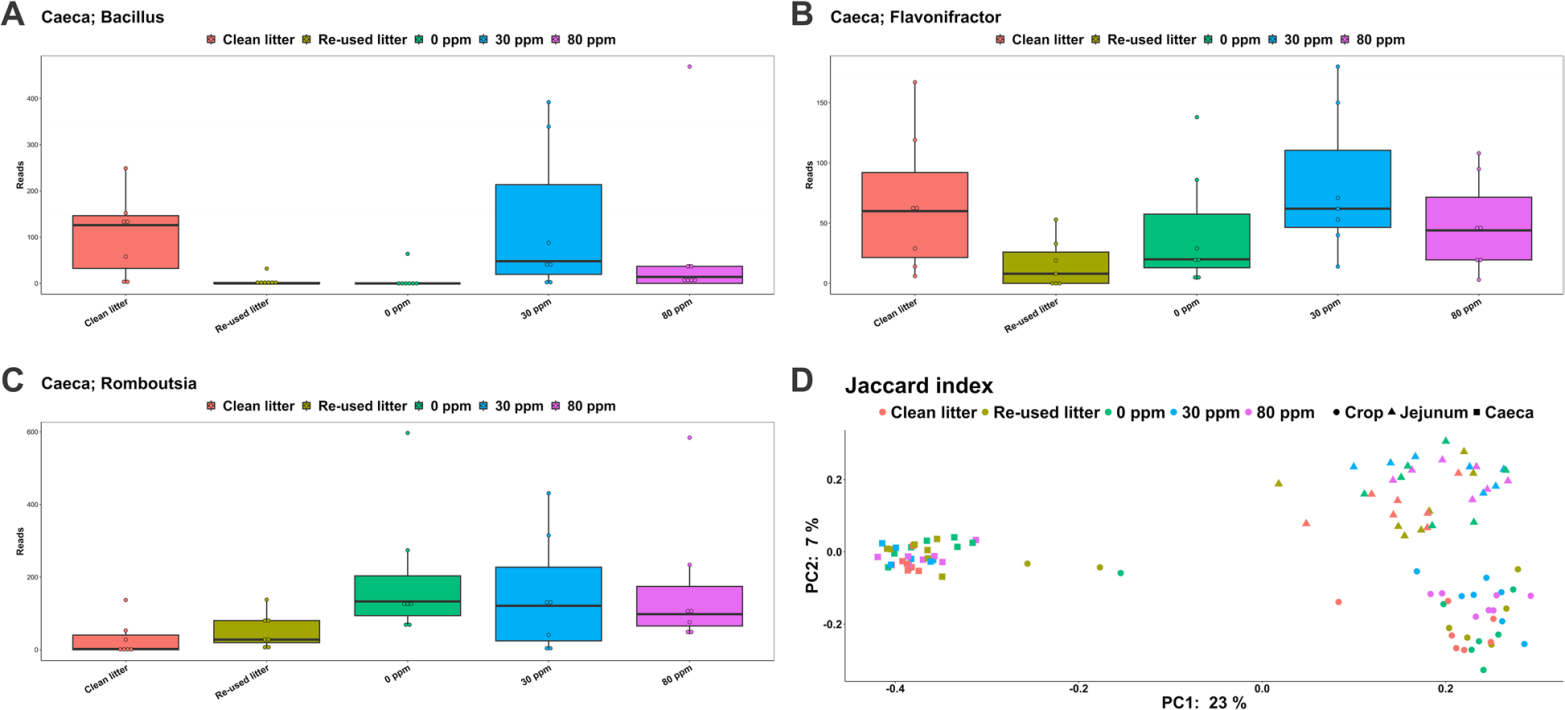
Read number of *Bacillus* (**A**), *Flavonifractor* (**B**) and *Rombustia* (**C**) found in the caecal lumen. **D** PCoA plot representing the distances between the samples calculated through the Jaccard index. Different colors represent the different experimental conditions, whereas different shapes depict the different gut locations analyzed

Shannon diversity (Fig. 3D) in crop and in jejunum did not vary between the five experimental conditions (3.3 ± 0.6 and 2.9 ± 0.7, in total, respectively). The caecal Shannon index in CLC (5.7 ± 0.2) tended to be greater (*P* = 0.071) than for RLC (5.4 ± 0.3), however no significant differences were found when compared to 0 ppm (5.5 ± 0.2), 30 ppm (5.6 ± 0.4) and 80 ppm (5.5 ± 0.4). Crop richness was 101.1 ± 60.9 in RCL, 75.6 ± 23.7 in CLC and 80.7 ± 23.5 in the PAA interventions. Whilst richness in the jejunum averaged 54 ± 25.5 across the treatments, caecal richness in 30 ppm (188.6 ± 38) tended to be greater (*P* = 0.064) than for RLC (165.1 ± 44.6). *Bacillus* (Fig. 4A) tended to be increased in 30 ppm and CLC compared to RLC (*P* < 0.05, *Q* = 0.1), whilst *Flavonifractor* (Fig. 4B) tended to be more represented in 30 ppm compared to RLC (*P* < 0.05, *Q* = 0.1). On the other hand, *Rombustia* (Fig. 4C) in CLC were decreased compared to 0 ppm and 80 ppm (*P* < 0.05, *Q* < 0.05). Moreover, both *Anaerostipes* and *Escherichia/Shigella* were decreased in CLC compared to 0 ppm (*P* < 0.05, *Q* = 0.07), whilst *Blautia* was more abundant in CLC than for 0 ppm (*P* < 0.05, *Q* = 0.07). *Anaerostipes* was also, together with *Ruminococcus* (*gauvreauii* group), less represented in 80 ppm compared to 0 ppm (*P* < 0.05, *Q* = 0.09), whilst *Bacillus* and *Clostridia* UCG-014 were reduced in 0 ppm compared to 30 ppm (*P* < 0.05, *Q* = 0.09).

Principal coordinate analysis (PCoA) based on Bray–Curtis and Jaccard distances (β-diversity, Fig. 4D) revealed that samples were clustered into three distinct groups, with crop and jejunum somewhat overlapping. Jaccard metrics indicated that crop RLC communities tended to be distant from CLC (*P* = 0.058, *Q* = 0.076), whilst the latter was different compared to 30 ppm (*P* < 0.05, *Q* < 0.05). CLC communities in the jejunum were showed less similarities when compared to 30 ppm (*P* < 0.05, *Q* < 0.05), and caecal communities in 0 ppm were significantly distant from both CLC and 30 ppm (*P* < 0.05, *Q* < 0.05). Likewise, according to the Bray–Curtis distances, RCL communities in jejunum were significantly distant compared to 30 ppm (*P* < 0.05, *Q* < 0.05), whilst caecal CLC communities were significantly distant from both RLC, 0 ppm and 30 ppm (*P* < 0.05, *Q* < 0.05).

### Caecal AMR gene relative abundance

**Fig. 5:**
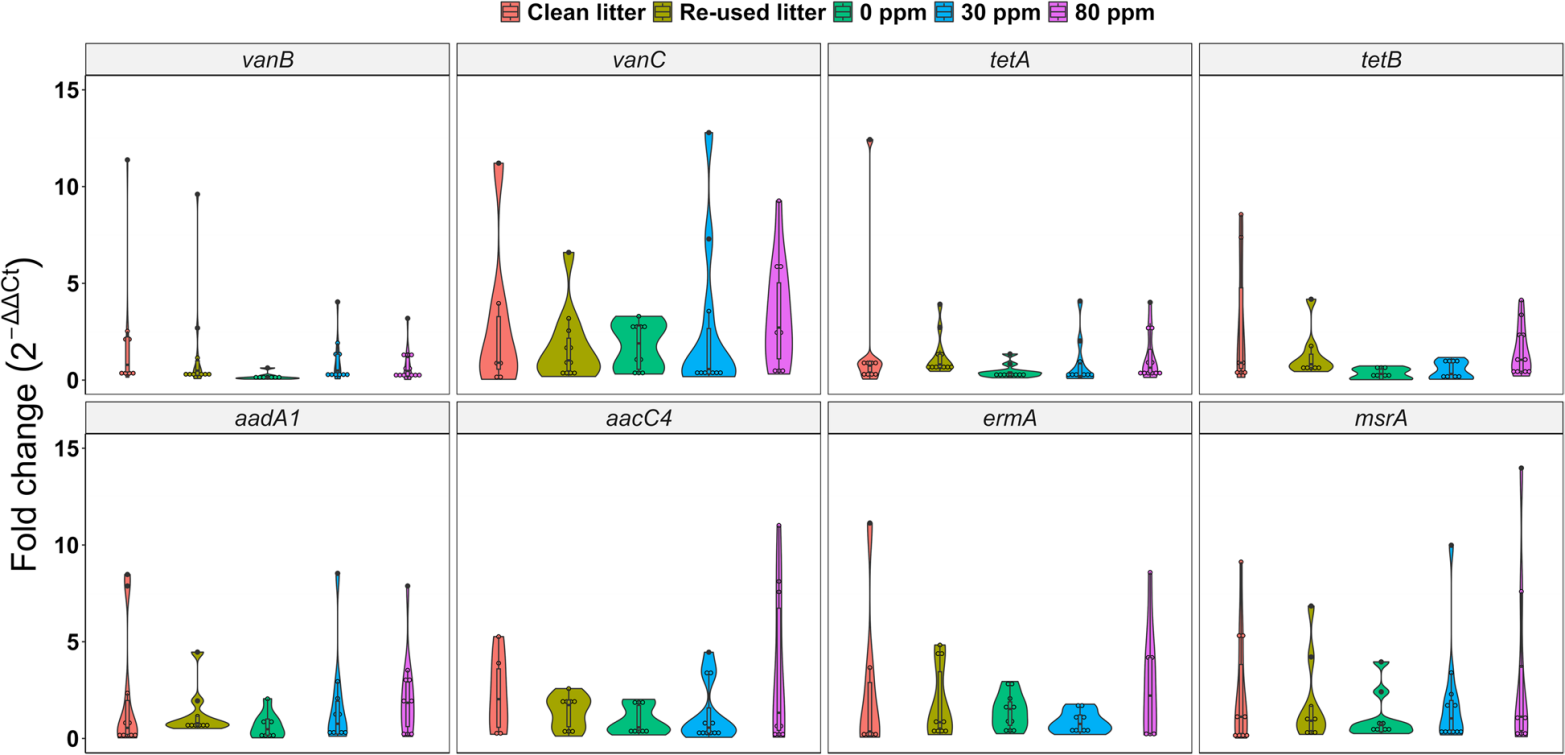
Differential abundance of eight AMR genes throughout the different experimental conditions

Of the 20 AMR genes analyzed, only 8 (*vanB, vanC, tetA, tetB, aadA1, aacC4, ermA* and *msrA*) were found to be present in all the samples analyzed and therefore allowing statistical analysis for differential relative abundance (i.e., not expression) as discussed below (Fig. 5). *SHV, ermC* and *QnrB-8* were randomly found (i.e., Ct < 40) only in a few samples, thus not allowing a proper comparison between the treatments, whilst *aphA6, mecA, ctx-M-1, ctx-M-9, OXA-48, QnrD, aprm, ccrA* and *ereB* were not found (i.e., no Ct) in any of the samples analyzed.

Fold change relative abundance of *vanB* was greater for CLC (2.3 ± 3.5) than for 0 ppm (0.2 ± 0.2; *P* < 0.05), also supported by the contrast statement for CLC compared to the rest of the experimental conditions (*P* = 0.08). A significant linear relationship, F(4, 48) = 2.87 (*P* < 0.05) was found between *vanC* relative abundance and PAA level. Furthermore, *vanC* in CLC (17.3 ± 29.1) was significantly greater than RLC (1.7 ± 1.9), 0 ppm (1.8 ± 1.2), 30 ppm (2.6 ± 4) and 80 ppm (3.3 ± 2.7; all *P* < 0.05), also corroborated by a significant contrast for CLC against the rest of the conditions (*P* < 0.05). Similarly, the same contrast revealed a significant difference between *tetA* relative abundance in CLC (4.6 ± 9.3) and the rest of the experimental conditions (*P* < 0.05). Indeed, the former tended to be greater than RLC (1.3 ± 1.1; *P* = 0.07) and 80 ppm (1.2 ± 1.2, *P* = 0.06), whilst it was significantly greater than 0 ppm (0.5 ± 0.4) and 30 ppm (0.8 ± 1.3; *P* < 0.05 for both). Treatment had significant effect on *tetB* abundance (F(4, 31.6) = 3.1,* P* < 0.05), mainly due to the differences between CLC and the rest of the treatments (contrast *P* < 0.05). Indeed, *tetB* level in CLC (2.9 ± 3.4), was greater than 0 ppm (0.3 ± 0.3) and 30 ppm (0.5 ± 0.5; both *P* < 0.05) and tended to be greater than RLC (1.3 ± 1.2, *P* = 0.08) and 80 ppm (1.5 ± 1.3, *P* = 0.07). No significant differences were found in the abundance levels of *aadA1*. Whilst *aacC4* LMM showed a trend towards treatment effect (F(4, 41) = 2.1, *P* = 0.1), it was clear that the most noticeable change was towards an increased abundance in 80 ppm (3.5 ± 4), which was significantly greater than RLC (1.4 ± 0.9), 0 ppm (0.9 ± 0.7) and 30 ppm (1.3 ± 1.5; *P* < 0.05 for all), although with rather similar levels to CLC (2.3 ± 2, *P* = 0.31). These findings were supported by both the linear contrast (30 ppm vs. 80 ppm, *P* < 0.05) and by the quadratic one indicating an optimum at 80 ppm (*P* < 0.05). This was similar to what found for *ermA*, whose 80 ppm abundance (6 ± 7.4) was significantly greater compared to RLC (1.8 ± 1.9), 0 ppm (1.4 ± 0.9) and 30 ppm (0.8 ± 0.6; *P* < 0.05 for all) but similar to CLC (4.6 ± 7, *P* = 0.5), although the latter tended to be greater than 30 ppm (*P* = 0.08). Whilst the LMM showed a trend for treatment effect (F(4, 44) = 2.5, *P* = 0.06), both the 30 ppm vs. 80 ppm contrast and the quadratic one with optimum at 80 ppm (both *P* < 0.05) corroborated the greater significant levels found for 80 ppm. Finally, a similar scenario was found for *msrA* relative gene abundance, where levels associated to 80 ppm (8.2 ± 11.8) were greater than those in CLC (2.4 ± 3), RLC (1.8 ± 2.2), 0 ppm (1.1 ± 1.3) and 30 ppm (2.9 ± 4.5; *P* < 0.05 for all), again corroborated both by the quadratic (optimum at 80 ppm) statement and the 30 pp vs. 80 ppm linear one (*P* < 0.05 for both). Moreover, the LMM showed a trend for treatment effect (F(4, 45.5) = 2.5, *P* = 0.06), whilst a trend was also found for 0 ppm vs. PAA treatment contrast analysis (*P* = 0.06).

### PAA influence on performance and luminal pH

A total mortality of 8 birds was recorded over the 28-day study (CLC = 1, RLC = 3, 0 ppm = 0, 30 ppm = 1 and 80 ppm = 3). The experimental conditions significantly affected bird performance (Table 3). At placement (d 0), average body weight was 40 ± 0.003 g, which did not differ between experimental treatments. However, LMM revealed a significant effect of the experimental treatments on log BWG (F(4, 57) = 3.8, *P* < 0.05), a significant time effect (F(3,1083) = 28,127, *P* < 0.001) and a significant interaction between experimental conditions and time (F(12, 1083) = 2.8, *P* < 0.05). Specifically, at d 7, 14 and 21, BWG in the RLC group was smaller than both 30 ppm and 80 ppm (*P* < 0.05), whilst also showing a lower trend compared to 0 ppm at d 7 (*P* = 0.07), d 14 (*P* = 0.06) and compared to 30 ppm at d 28 (*P* = 0.07). BWG in the CLC group was smaller than 0 ppm, 30 ppm and 80 ppm at d 14, 21 and 28 (*P* < 0.05). Contrast statement analysis confirmed that RLC was different from 0, 30 and 80 ppm at d 7, 14 and 21 (*P* < 0.05) and d 28 (*P* = 0.09), whereas CLC was different from the rest of the experimental conditions at d 14, 21 and 28 (*P* < 0.05).

**Table 3:** Performance and pH observed through the different time intervals and gut locations amongst the five experimental conditions

**Interval/Location**	**CLC**	**RLC**	**0 ppm**	**30 ppm**	**80 ppm**
Bird-level body weight, kg					
d 0 (*n* = 375)	0.041 ± 0.003	0.04 ± 0.003	0.04 ± 0.002	0.04 ± 0.003	0.04 ± 0.003
Bird-level body weight gain, kg					
d 0–7 (*n* = 373)	0.11 ± 0.03	0.11 ± 0.03^A,a^	0.11 ± 0.03^b^	0.12 ± 0.03^B^	0.12 ± 0.03^B^
d 0–14 (*n* = 371)	0.33 ± 0.08^A^	0.33 ± 0.08^C,c^	0.36 ± 0.07^B,d^	0.37 ± 0.07^B,D^	0.36 ± 0.07^B,D^
d 0–21 (*n* = 369)	0.72 ± 0.18^A^	0.76 ± 0.17^C^	0.79 ± 0.14^B^	0.82 ± 0.14^B,D^	0.82 ± 0.12^B,D^
d 0–28 (*n* = 367)	1.28 ± 0.29^A^	1.37 ± 0.27^c^	1.41 ± 0.22^B^	1.45 ± 0.22^B,d^	1.42 ± 0.18^B^
Pen-level mortality-corrected feed conversion ratio					
d 0–7 (*n* = 75)	1.12 ± 0.06^a^	1.17 ± 0.05^b,C^	1.11 ± 0.07^D^	1.09 ± 0.08^D^	1.08 ± 0.08^D^
d 0–14 (*n* = 75)	1.2 ± 0.06^A^	1.19 ± 0.02^c^	1.17 ± 0.04	1.14 ± 0.05^B,d^	1.17 ± 0.03
d 021 (*n* = 75)	1.3 ± 0.08^A^	1.27 ± 0.06^c^	1.3 ± 0.03^E^	1.27 ± 0.07^ g^	1.22 ± 0.1^B,d,F,h^
d 0−28 (*n* = 75)	1.38 ± 0.1^A^	1.31 ± 0.11^B,C^	1.37 ± 0.03^D,E^	1.34 ± 0.08	1.31 ± 0.11^B,F^
Bird-level luminal pH					
Ileum (*n* = 148)	6.6 ± 0.9^A^	6.6 ± 0.8^C^	6.9 ± 0.7	6.8 ± 0.8^E^	7.2 ± 0.5^B,D,F^
Caeca (*n* = 149)	6.2 ± 0.6	6.2 ± 0.7	6.0 ± 0.7	6.0 ± 0.7	6.0 ± 0.7
Colon (*n* = 147)	6.6 ± 0.9	6.6 ± 1	6.7 ± 1	6.5 ± 0.8	6.6 ± 0.9

*CLC* Clean litter control, *RLC* Re-used litter control; *0 ppm* 0 mg/kg of PAA control, *30 ppm* 30 mg/kg of PAA treatment, *80 ppm* 80 mg/kg of PAA treatment. Different uppercase superscripts in the same row indicate statistically significant differences (*P* < 0.05), whilst lower case superscripts indicate 0.05 < *P* < 0.10, as calculated via Satterthwaite's method applied on the output of the linear mixed model

Similarly, LMM revealed a significant effect of the experimental treatments (F(4, 56.5) = 2.8, *P* < 0.05), time (F(3, 210) = 258.7, *P* < 0.05) and their interaction (F(12, 210) = 2.8, *P* < 0.05) on MFCR, with the quadratic contrast indicating a possible optimum MFCR level (i.e., lower than other conditions) in 80 ppm (*P* < 0.05). Indeed, MFCR in the 80 ppm group was lower than 0 ppm (*P* < 0.05) and tended to be lower than 30 ppm at d 21 (*P* = 0.07), whereas it was lower than both 0 ppm and CLC at d 28 (*P* < 0.05). Moreover, MFCR in the CLC group tended to be lower than RLC at d 7 (*P* = 0.05), however the former was greater than 30 ppm at d 14, 80 ppm at d 21 and both RLC and 80 ppm at d 28 (all *P* < 0.05). MFCR in the RLC group was greater compared to 0 ppm, 30 ppm and 80 ppm at d 7 (*P* < 0.05), tended to be greater than 30 ppm at d 14 (*P* = 0.07), greater than 80 ppm at d 21 (*P* < 0.05) and smaller than CLC and 0 ppm at d 28 (*P* < 0.05). Contrast analysis confirmed that MFCR in RLC was greater than 0 ppm, 30 ppm and 80 ppm at d 7 (*P* < 0.05), that MFCR in the 0 ppm group was greater than the PAA treatments both at d 21 and 28 (*P* < 0.05), with a greater trend in 30 ppm compared to 80 ppm at d 28 (*P* = 0.07), and that MFCR in the CLC group was greater than the rest of the experimental conditions at d 21 (*P* = 0.07) and d 28 (*P* < 0.05).

Contrast statements revealed that ileal luminal pH tended to be lower for CLC compared to the reused litter treatments (*P* = 0.06), was significantly lower for the RLC than for the combined 0pmm, 30 ppm and 80 ppm treatment (*P* < 0.05), whilst also observing dose effect with greater pH in 80 ppm compared to smaller doses (*P* < 0.05). Indeed, whilst the LMM indicated a clear effect of the experimental conditions on the ileum pH (F(4, 66) = 3.2, *P* < 0.05), CLC, RLC and 30 ppm treatments were significantly lower than 80 ppm (*P* < 0.05). On the other hand, no significant differences were found in caecal and colonic luminal pH between the experimental treatments.

## Discussion

Finding new alternatives to antibiotics is a primary strategy to decrease the AMR burden in different sectors. This is highly relevant for poultry production, which is directly associated to the spread of AMR bacteria into both the environment and as a risk for the consumer. In this study, the proposed alternative PAA, administered via the hydrolysis of RLPO encapsulated SP and TAED, led to targeted decrease of some microbial communities in the jejunum which possibly further triggered the selection of beneficial genera in the caeca, especially for birds at the 30 ppm treatment. We reported a noticeable difference in the effect produced by the two levels of inclusion administered to the birds, with a possible optimum at 30 mg/kg of PAA instead of 80 mg/kg for a number of readouts. PAA is a well-known broad-spectrum antimicrobial agent that does not release harmful products and is effective towards both Gram-positive and Gram-negative bacteria in less than five min and at less than 100 mg/kg [45]. Its main mode of action is due to its oxidizing properties towards organic compounds [46], therefore compromising bacterial cell integrity and leading to denaturation of proteins and other intracellular metabolites detrimental to bacterial cell survival [13].We previously reported that PAA administered in water via unencapsulated precursors had a noticeable effect in the most proximal part of the gut, leading to a reduction of *Lactobacillus* in the crop of broilers [47]. The main effect recorded through the present study was a reduced abundance of Firmicutes in the jejunum, which seems to suggest a shift of PAA formation to the more distally located jejunum, likely due to the RLPO encapsulation. Previous studies indicated an increased Firmicutes abundance led by PAA during anaerobic dark fermentation of waste activated sludge [48], whose differences with our findings might reflect possible different PAA modes of action towards different bacterial targets depending on the environment and the relative compositional characteristics of the rest of the community. Amongst the Firmicutes, *Lactobacillus* abundance was decreased in the 30 ppm group, whilst *Enterococcus* and *Staphylococcus* had an opposite trend, also accompanied by an increase in *Escherichia/Shigella.* It is worth noticing how similar trends of these four genera were also found in the crop, although a significant reduction in Firmicutes was only visible in the jejunum, likely pointing to a peak of PAA generated in the more distal gut compartment. The postulated more distal release of precursors in the jejunum is compatible to the drug release dynamics associated with RLPO particles, as they should ensure time-controlled, pH independent release.

The PAA breakdown into peroxide and acetic acid rather than into potential harmful bioproducts is amongst the most attractive features of PAA as an antimicrobial alternative, especially for its use in vivo. Indeed, not only peroxide could assist the broad-spectrum antimicrobial action, but acetate could also function as prebiotic [14]. Our findings support this view; *Flavonifractor* is a known butyrate producer via using the butyryl-CoA: acetate CoA transferase pathway [49], in which exogenous acetate is used to produce acetyl-CoA and butyrate [50]. Therefore, it can be hypothesized that the acetate produced via SP and TAED hydrolysis could elicit the growth of acetate-consumers, with beneficial effects for the host [51]. However, the relative abundance of other acetate-consumers butyrate-producers did not differ between the experimental treatments, such as in the case of *Faecalibacterium* [52], whose relative abundance was similar in RLC and 30 ppm or 80 ppm and if anything, greater in abundance for the CLC treatment. In general, both *Flavonifractor* as a butyrate producer [53] and *Bacillus*, commonly used as a probiotic [54, 55] could explain the ameliorated performance associated with the 30 ppm group at d 28. Although *Bacillus* was also somewhat more abundant in CLC, therefore the increased performance observed in the 30 ppm group was most likely due to more complex multi-factorial compositional changes, possibly leading to specific host-microbiota interactions.

It must be observed that on average, the BW recorded throughout the study was somewhat inferior to the breed performance objectives [40], which may very well be the consequence of mash feeding rather than crumbs and pellets. Indeed, birds from all the treatments ended on average 13.6% below Ross 308 expected body weight at d 28. Nevertheless, at d 28, BW of birds in the 30 ppm group was only 9.8% (162 g) lower than performance objectives, followed by 80 ppm (11.7%), 0 ppm (12.1%), RLC (14.3%) and CLC (19.9%). Beyond the positive effects on performance of the PAA treatment, with an optimum at 30 mg/kg level of inclusion, our results also showed both an apparent improved performance for the 0 ppm group and lowest performance for the CLC group compared to all the rest of experimental treatments. Eudragit® RLPO copolymer is mainly composed by ethyl acrylate, methyl methacrylate and a low content of methacrylic acid ester, unlikely connected to any added nutritional values. Therefore, it is plausible that the addition of particles within the feed influenced the feeding pattern of the chickens, functioning as an enrichment. This is not uncommon, as other authors have documented an increased interest towards colored feed [56, 57], which could therefore explain the augmented feed intake of birds presented with particles. This is further supported by these birds performing at same FCR indicating the greater BW is derived from greater intake. However, RLPO-driven compositional changes cannot be excluded; we observed some increase in *Rombustia* abundancy in 0 ppm and 80 ppm, and non-significantly for 30 ppm.

Litter management differs in production systems across the globe, with some of the countries changing the litter material at each cycle and some of them re-using it for more than one [17]. There is a degree of ambiguity whether such reused litter exposure is detrimental or beneficial, as the effect of litter management on broiler performance and gut microbiota seems to vary amid studies. In some instances, re-used litter was connected to lower body weight and feed intake [58], concurring with a higher rate of foot-pad lesions and lower welfare indicators [59], whilst other studies found no differences in performance between birds raised on clean or re-used litter [60]. Our results indicated consistently reduced performance associated with CLC, when compared to the rest of the treatments, which all involved re-used litter. These discrepancies could indicate that factors like litter provenience and storage time/conditions between growing cycles may influence response to litter exposure. It ought to be acknowledged that although we do not have microbial compositional information of litter at d 0, caecal taxonomical differences were observed at trial end between CLC and the other treatments. Relative to the latter, CLC abundance of *Anaerostipes*, *R. torques* group, *Bifidobacterium*, *Lactobacillus*, unassigned and uncultured *Lachnospiraceae* was decreased, whilst that of *Faecalibacterium* and *Blautia* was increased. We also found that *Escherichia* and *Lactococcus* were increased in the crop of the CLC group compared to the rest of the experimental treatments, whilst *Staphylococcus* was decreased, which was also decreased in the jejunum of CLC birds, whilst *Enterococcus* was increased. Moreover, some studies indicate a correlation between low microbial diversity and a reduced microbial ability to maintain gut homeostasis [61], although this could not explain the apparent lower performance levels found in CLC compared to the other experimental conditions that at the same time were connected to a reduced Shannon index. Interestingly, richness and qPCR results were in apparent contrast, as the reduction of concentration in ileum in 0 ppm and 80 ppm found via qPCR did not agree with the increased richness in the caecal content of birds in the 80 ppm group.

Genes conferring resistance to some aminoglycosides (*aphA6*), beta-lactams (*SHV*, ccrA, *ctx-M-1*, *ctx-M-9*, *mecA* and *OXA-48*), some quinolones and fluoroquinolones (*QnrD*, *QnrB-8* and *aprm*), some macrolides, lincosamides and streptogramins (*ermC*)*,* and erythromycin (*ereB*) were not detected in any of the samples analyzed. Genes *vanB* and *vanC*, which are associated to vancomycin-resistant *Enterococci* [62], were more abundant in CLC, although the relative abundance of *Enterococcus* throughout the samples did not vary significantly, possibly indicating that other genera acting as reservoir could have been differentially abundant through the samples. The tetracycline efflux pump encoded by *tetA* [63] and *tetB* [64] are found in many bacteria. As such, their increased abundance in CLC could be explained by the establishment of different resistant microbes on the re-used litter. Moreover, the relative abundance of *tetA* and *tetB* was lower in 30 ppm compared to CLC. Both *aadA1* and *aacC4* confer resistance to aminoglycoside antibiotics and have been identified in several bacterial species [65, 66], although we did not observe peculiar differences in their relative abundance throughout the five experimental conditions. Interestingly, even thought we did not find *ermC* throughout our samples, *ermA,* responsible for ribosomal binding site alteration conferring resistance to macrolides, lincosamides and streptogramins [67], was found differentially abundant throughout the conditions and especially correlated with higher abundance within the 80 ppm group. This, together with the other macrolide resistance gene *msrA* [68], possibly indicated a potential action of PAA towards species lacking in these AMR.

## Conclusions

The present study demonstrated that PAA could be used as a broad-spectrum antimicrobial alternative in vivo in broiler birds, connected with low host toxicity, optimum level of inclusion at 30 mg/kg under the current trial conditions and mainly targeting Firmicutes. We observed a reduction in bacterial concentration in the jejunum associated with both the 0 ppm and 80 ppm group, but in a similar fashion, however not statistically significant in the 30 ppm group. This could indicate that the particle hydrolysis could have mainly been occurring in the jejunum, however only treatments delivering PAA were linked to a noticeable effect towards modification of the microbial communities, likely indicating that the empty particles were lacking of broad-spectrum antimicrobial activity. These results also demonstrated the efficacy of the RLPO encapsulation of the precursors SP and TAED in bypassing the upper gut and delivering active molecules and their benefits more distally, i.e., from the jejunum to the caeca. Our results also possibly pointed to not only a low toxicity of the by products of PAA formation, but also to a possible probiotic effect, hypothesized to be exerted by acetate, likely able to elicit the growth of some probiotic strains such as *Flavonifractor.* Further studies are recommended in order to identify optimal active concentration to be used in vivo as our 80 ppm levels may select for macrolide resistance. We observed a correlation between PAA administration and increased growth performance. In particular, we observed an increased BWG in both the 30 ppm and 80 ppm groups in the first three weeks, when compared to the control on re-used litter, and a trend in BWG increase for the 30 ppm group when compared to the same control through the 28 d. This could have possibly indicated that the microbial modifications introduced by PAA as a broad-spectrum antimicrobial alternative could have been beneficial for the host, especially during the first three weeks of the animal study. This study also contributed to elucidating beneficial consequence of broiler production on reused litter. Throughout our animal study, we found that clean litter correlated with lower performance and likely increased relative abundance of some resistance genes. Therefore, further studies are recommended, to correlate the effect of re-used litter when exposed to different storage conditions and difference provenience.

## Supplementary Information

**Additional file 1.** Ingredients composition and nutrition levels of bespoke commercial diets.

**Additional file 2.** Taxonomy at phylum and genus levels.

## Data Availability

The datasets used and/or analyzed during the current study are available from the corresponding author on reasonable request.

## Abbreviations

AMR: Antimicrobial resistance
MDRO: Multi-drug resistant organisms
PAA: Peracetic acid
PPC: Positive PCR control
ppm: Parts per million
qPCR: Quantitative real polymerase chain reaction
RLPO: Eudragit™ ammonio methacrylate copolymer type A
SP: Sodium percarbonate
TAED: Tetraacetylethylenediamine
