# Supplementary material for: Encapsulated peracetic acid as a valid broad-spectrum antimicrobial alternative, leading to beneficial microbiota compositional changes and enhanced performance in broiler chickens
Source: J Anim Sci Biotechnol. 2023 Jun 9;14:83. doi: 10.1186/s40104-023-00881-w (PMC10251604; doi:10.1186/s40104-023-00881-w)
Supplement: Supplementary file 1 — Additional file 1. Ingredients composition and nutrition levels of bespoke commercial diets. [file 40104_2023_881_MOESM1_ESM.docx]

|  | STARTER | GROWER |
| --- | --- | --- |
| Raw Material | % | % |
| Barley | 10.403 | 8.300 |
| Wheat | 50.000 | 55.00 |
| Soya Ext Hipro | 26.000 | 23.00 |
| Full fat Soya Cherwell | 5.000 | 5.000 |
| L Lysine HCl | 0.400 | 0.300 |
| DL-methionine | 0.400 | 0.350 |
| L-threonine | 0.150 | 0.150 |
| Soya Oil | 4.000 | 4.500 |
| Limestone Trucal 52 | 1.250 | 1.250 |
| Monocalcium phosphate | 1.500 | 1.250 |
| Salt | 0.250 | 0.250 |
| Sodium bicarbonate | 0.150 | 0.150 |
| Broiler trials Supp. (Premix) ^1^ | 0.400 | 0.400 |
| Maxiban G160 Premix | 0.0625 | 0.0625 |
| Ronozyme WX | 0.020 | 0.020 |
| Ronozyme P 5000 (CT) | 0.015 | 0.015 |
| Total | 100 | 100 |
| Nutrients - Calculated Analysis %, unless otherwise stated | | |
| Fat (ether extract) | 6.39 | 6.85 |
| Protein | 21.84 | 20.64 |
| Fibre | 3.08 | 3.02 |
| Ash | 6.02 | 5.68 |
| Metabolisable energy (MJ/Kg) | 12.73 | 13.04 |
| Total lysine | 1.43 | 1.28 |
| Available lysine | 1.33 | 1.19 |
| Methionine | 0.69 | 0.62 |
| Total methionine and cysteine | 1.03 | 0.95 |
| Threonine | 0.91 | 0.86 |
| Tryptophan | 0.25 | 0.23 |
| Calcium | 0.95 | 0.91 |
| Phosphorus | 0.73 | 0.66 |
| Available phosphorus | 0.48 | 0.42 |
| Salt | 0.30 | 0.30 |
| Sodium | 0.17 | 0.17 |
| Vit A IU | 13.50 | 13.5 |
| Vit D3 IU | 5.00 | 5.00 |
| Vit E IU | 100 | 100 |
| Notes: ^1^Per kilogram premix: vit. A: 2.4 MIU; vit. D3: 1 MIU; vit. E: 10,000 IU; vit. K3: 600 mg/kg; vit. B1: 400 mg; vit. B2: 1,400 mg; pantothenic acid: 3,000 mg; nicotinic acid 10,000 mg; vit. B6: 1,000 mg; vit. B12: 3,000 ug; folic acid: 200 mg; biotin: 40 mg; Ca: 31.9%; P: 0.026%; Na: 0.071%; Cl: 0.023%; K: 0.035%; S: 1.114%; Fe (sulphate): 16,000 mg; Cu (sulphate): 2,000 mg; Zn (oxide): 16,000 mg; Mn (oxide): 20,000 mg; I (Ca-iodate): 200 mg; Co (carbonate) 100 mg; Se (Na-selenite): 40 mg; Mo (Na-molybdenum): 100 mg. | | |
